# Study protocol for the optimisation, feasibility testing and pilot cluster randomised trial of Positive Choices: a school-based social marketing intervention to promote sexual health, prevent unintended teenage pregnancies and address health inequalities in England

**DOI:** 10.1186/s40814-018-0279-3

**Published:** 2018-05-23

**Authors:** Ruth Ponsford, Elizabeth Allen, Rona Campbell, Diana Elbourne, Alison Hadley, Maria Lohan, G. J. Melendez-Torres, Catherine H. Mercer, Steve Morris, Honor Young, Chris Bonell

**Affiliations:** 1 London School of Hygiene and Tropical Medicine, 15-17 Tavistock Place, London, WC1H 9SH UK; 2 London School of Hygiene and Tropical Medicine, Keppel Street, London, WC1E 7HT UK; 3 University of Bristol, 39 Whatley Road, Bristol, BS8 2PS UK; 4 University of Bedfordshire, University Square, Luton, LU1 3JU UK; 5 Queens University Belfast, University Road, Belfast, BT7 1NN UK; 6 Cardiff University, 1-3 Museum Place, Cardiff, CF10 3BD UK; 7 University College London, Gower Street, London, WC1E 6BT UK; 8 University College London, 1-19 Torrington Place, London, WC1E 7HB UK

**Keywords:** Teenage pregnancy, Sexual health, SRE, RSE, School intervention, Adolescent, Cluster randomised trial

## Abstract

**Background:**

Since the introduction of the Teenage Pregnancy Strategy (TPS), England’s under-18 conception rate has fallen by 55%, but a continued focus on prevention is needed to maintain and accelerate progress. The teenage birth rate remains higher in the UK than comparable Western European countries. Previous trials indicate that school-based social marketing interventions are a promising approach to addressing teenage pregnancy and improving sexual health. Such interventions are yet to be trialled in the UK. This study aims to optimise and establish the feasibility and acceptability of one such intervention: Positive Choices.

**Methods:**

Design: Optimisation, feasibility testing and pilot cluster randomised trial.

Interventions: The Positive Choices intervention comprises a student needs survey, a student/staff led School Health Promotion Council (SHPC), a classroom curriculum for year nine students covering social and emotional skills and sex education, student-led social marketing activities, parent information and a review of school sexual health services.

Systematic optimisation of Positive Choices will be carried out with the National Children’s Bureau Sex Education Forum (NCB SEF), one state secondary school in England and other youth and policy stakeholders.

Feasibility testing will involve the same state secondary school and will assess progression criteria to advance to the pilot cluster RCT.

Pilot cluster RCT with integral process evaluation will involve six different state secondary schools (four interventions and two controls) and will assess the feasibility and utility of progressing to a full effectiveness trial.

The following outcome measures will be trialled as part of the pilot:

1. Self-reported pregnancy and unintended pregnancy (initiation of pregnancy for boys) and sexually transmitted infections,
2. Age of sexual debut, number of sexual partners, use of contraception at first and last sex and non-volitional sex
3. Educational attainment

The feasibility of linking administrative data on births and termination to self-report survey data to measure our primary outcome (unintended teenage pregnancy) will also be tested.

**Discussion:**

This will be the first UK-based pilot trial of a school-wide social marketing intervention to reduce unintended teenage pregnancy and improve sexual health. If this study indicates feasibility and acceptability of the optimised Positive Choices intervention in English secondary schools, plans will be initiated for a phase III trial and economic evaluation of the intervention.

**Trial registration:**

ISRCTN registry (ISCTN12524938. Registered 03/07/2017).

**Electronic supplementary material:**

The online version of this article (10.1186/s40814-018-0279-3) contains supplementary material, which is available to authorized users.

## Background

### Teenage pregnancy and sexual health

Between 1998 and 2015, a period which included the 1999–2010 implementation of the Labour government’s Teenage Pregnancy Strategy (TPS), England’s under-18 conception rate has fallen by 55% [1]. Following the conclusion of the strategy, a continued focus on prevention is needed to maintain progress and reduce disparities in conception rates between different parts of the country. Data from 2017 indicate that there is a sixfold difference in the under-18 conception rate between local authorities (LAs) and 60% of LAs have at least one electoral ward with a significantly higher rate than the average for England [2]. The teenage birth rate remains higher in England and Wales than comparable Western European countries [3]. Even after controlling for prior disadvantage, teenage pregnancy is associated with adverse medical, social, educational and economic outcomes for both mothers [4–6] and children [7, 8]. Teenage pregnancy is a symptom of and contributes to the maintenance of health inequalities [9]. In 2006, it was estimated that teenage pregnancy cost the NHS £63 million per year [10]. HIV and other sexually transmitted infections (STI) disproportionally affect young adults and cost the NHS large sums [11, 12]. However, effective prevention saves money. Return on investment analysis of the Teenage Pregnancy Strategy, for example, calculated that for every £1 spent, £4 was saved [13].

### Social marketing interventions to prevent teenage pregnancy

A recent systematic review of social marketing interventions to reduce teenage pregnancy examined studies of interventions embracing social marketing elements regardless of whether these were explicitly termed ‘social marketing’ in their description [14]. Heterogeneity precluded meta-analysis but narrative synthesis concluded this was a promising approach to addressing unintended teenage pregnancy.

‘Safer Choices’ and the ‘Children’s AIDS Society (CAS) Carrera’ Program were two effective social marketing interventions identified in the above review. Safer Choices is a school-based intervention involving a school health promotion council coordinating intervention activities, a classroom-based sexual health curriculum, student-led social marketing campaigns and information for parents. A randomised controlled trial (RCT) of this intervention in the US reported reduced unprotected last sex and reduced numbers of partners with whom unprotected sex occurred but did not measure effects on pregnancy [15–17]. The ‘CAS Carrera’ Program is an after-school intervention providing careers, academic, arts, sports and life-skills sessions and sexual health services. An RCT of this intervention in New York City reported fewer pregnancies and delayed sexual debut among girls [18]. An attempted replication trial in other US locations reported no such reductions, reportedly due to poor fidelity [19]. The Gatehouse project, although not cited in the above review, is a further intervention that adopts social marketing principles and was found to be effective in postponing age of sexual debut. The Gatehouse project is a school-based intervention which includes a student needs survey and classroom-based curriculum addressing social and emotional learning. Although primarily addressing mental health, an RCT in Australian high schools reported participants’ increased age of sexual debut, but did not measure impacts on teenage pregnancy explicitly [20].

School-based social marketing initiatives to prevent unintended teenage pregnancy and promote sexual health have not been trialled in the UK. Our study aims to optimise, feasibility test and pilot trial in English secondary schools a whole-school social marketing intervention called ‘Positive Choices’. Positive Choices is informed by selected components from the Safer Choices, CAS Carrera and Gatehouse programmes and ‘whole-school’ approaches to health improvement found to be effective in addressing a range of health risk behaviours, including those related to teenage pregnancy and sexual health [21, 22]. The Positive Choices intervention involves multiple components comprising a student needs survey, a student/staff led School Health Promotion Council, a classroom curriculum addressing social/emotional skills and sex education, student-led social marketing, parent information and a review of school sexual health services. The use of a student need survey to inform school coordination of the intervention by a student/staff council was informed by the Gatehouse intervention. The use of a school health promotion council, classroom curriculum and student-led social marketing was informed by the Safer Choices intervention. The intervention was informed by the CAS Carrera intervention not in terms of specific activities but more in terms of a focus on aiming to promote sexual health via a focus on developing positive skills and attitudes. In this study, our aim is not to assess the effects of Positive Choices, but to optimise the intervention with the National Children’s Bureau Sex Education Forum (NCB SEF)––a voluntary sector organisation that advocates for and provides resources to support the delivery of evidence and rights-based relationships and sex education in England––one state secondary school and other stakeholders, feasibility test and refine the intervention in the optimisation school, and conduct a pilot RCT and process evaluation in six schools to assess the feasibility and acceptability of the intervention and our trial methods. The study will determine the feasibility and utility of conducting a phase III trial of intervention effectiveness and cost effectiveness. Our research questions are outlined below.

### Research questions

1. Is it possible to optimise Positive Choices in collaboration with NCB SEF, a secondary school and other stakeholders?
2. Is it feasible and acceptable to implement each component of this intervention in the secondary school involved in optimisation and what refinements are suggested?
3. In light of a pilot RCT across six schools, is progression to a phase III trial justified in terms of pre-specified criteria?
4. Are secondary outcome and covariate measures reliable and what refinements are suggested?
5. With what rates are schools recruited to and retained in the trial?
6. What level of student reach does the intervention achieve?
7. What do qualitative data suggest in terms of intervention mechanisms and refinements to programme theory and theory of change?
8. How do contextual factors appear to influence implementation, receipt and mechanisms of action?
9. Are any potential harms suggested and how might these be reduced?
10. What sexual health-related activities occur in and around control schools?
11. Are methods for economic evaluation in a phase III trial feasible

## Methods

The study is 33 months long involving:

1. A facilitated, systematic optimisation of the Positive Choices intervention with the National Children’s Bureau Sex Education Forum (NCB SEF), a state secondary school and other youth and policy stakeholders––(April 2017–March 2018).
2. A formative feasibility assessment of intervention components in one secondary school and subsequent intervention refinement––(September 2017–August 2018).
3. An external pilot cluster RCT across six schools with integral process evaluation and study to assess the feasibility of economic evaluation—(May 2018–July 2019).

**Fig. 1:**
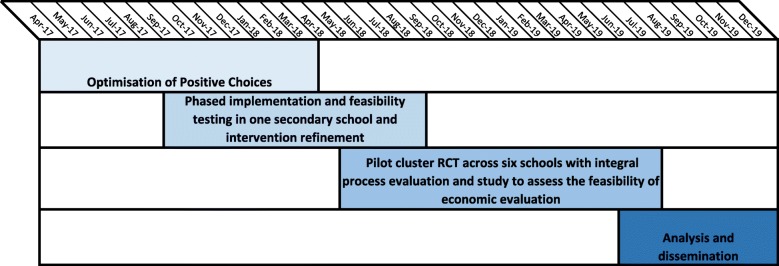
Overall study timeline

Figure 1 below illustrates the overall timeline for the study.

### Intervention

#### Intervention theory

**Fig. 2:**
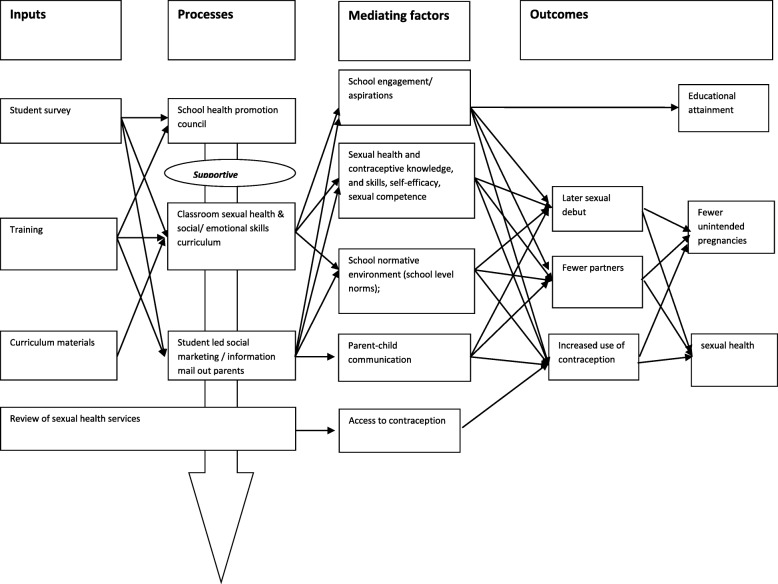
Logic model of Positive Choices

The Positive Choices programme theory is informed by social marketing principles, and has been developed with experts in this field, addressing the ‘4Ps’ [23–25]. Positive Choices will ‘sell’ consumers a **P**roduct *they* want (education on emotions and relationships) in an accessible **P**lace (school) at a low **P**rice (free to students), with **P**romotion to peers and parents (campaigns, parent information) addressing competing influences from peers, media etc. [26]. Our survey component enables our School Health Promotion Councils (with student involvement) to tailor provision in each school to local consumer priorities. The intervention theory of change (see Fig. 2) draws on those used in the interventions informing Positive Choices (Safer Choices, CAS Carrera, and the Gatehouse project).

Our theory of change will be further developed in the optimisation phase informed by models of school change, [27] social influence [28] and social cognitive theory [29] to address the following factors associated with reduced risk of teenage pregnancy and STIs and improved sexual health: sexual health knowledge, self-efficacy, skills and competence; communication with parents; school-wide social norms supporting positive relationships/sexual health. Informed by the social development model [30], we anticipate that the intervention will also lead to positive aspirations and school engagement, a further determinant of teenage pregnancy [31]. The review of sexual health services will also improve access to contraception. Although Positive Choices is a universal intervention, by addressing determinants of teenage pregnancy, such as lack of positive aspirations and school engagement, that are socially stratified, the intervention is intended to reduce sexual health inequalities.

#### Intervention components

Positive Choices is a manualised, whole-school social marketing intervention, delivered for one English academic year (September–July) in the feasibility testing and pilot trial phases. In a full trial, we expect the intervention to be delivered over two consecutive academic years. The intervention is intended to build on and augment rather than entirely replace existing school sex education and sexual health provision. Some flexibility is built into the programme so that schools can tailor intervention activities to the specific needs of their pupils.

The Positive Choices intervention comprises:

1. A student needs survey of year 8 students (aged 12–13) which will be used to enable each intervention components 3–6 below to be tailored to local priorities in each school.
2. A School Health Promotion Council (SHPC) which will comprise six staff/six students from different year groups who will review local needs data and use the data to tailor each intervention component 3–6 below to the school and will then coordinate in school delivery of the intervention.
3. A classroom curriculum that will address social/emotional skills (5-h class time per year) and sex education (5-h class time per year) delivered by school staff. The curriculum will be designed as a set of learning modules. Social and emotional skill modules will cover establishing respectful relationships in the classroom and the wider school, managing emotions, understanding and building trusting relationships, exploring others’ needs and avoiding conflict, and maintaining and repairing relationships. Sexual health modules will cover healthy relationships, negotiation and communication skills, positive sexual health, sexual risk reduction, contraception and local services. Informed by the needs-assessment data, School Health Promotion Councils will select in what order to deliver modules; whether to deliver within personal, social and health education (PSHE); tutor groups or integrated into other lessons (e.g. English) and whether to use our materials or existing materials if these conform to our curriculum.
4. Student-led social marketing facilitated by trained teachers and led by teams of 12–18 students per school. Campaigns may use social and other media, posters and events and will focus on healthy relationships, sexual and human rights, delayed sex and access to local services. Student social marketers will use data from the student needs survey to segment the student population based on multiple characteristics such as existing knowledge and attitudes to sexual health as well as cultural styles (e.g. hip-hop, skate) and peer group identifications (e.g. sporty boys, cool girls). The student social marketers will use such information to design social marketing campaigns which address the most important topics among the groups who need interventions most.
5. Parent information—three newsletters, two homework assignments per year addressing parent-child communication.
6. Consultancy on school sexual health services, which will involve an audit of available sexual health services in and around school, their accessibility for young people and how they are promoted within schools.

In the feasibility assessment phase, different intervention components will be delivered in different terms of one academic year. In the pilot, intervention components are implemented at the start of the academic year. In the feasibility testing and pilot trial phases, the curriculum element will be targeted at year 9 pupils (aged 13–14). In a full trial, it is envisaged that a classroom curriculum would be delivered to both year 9 (aged 13–14) and year 10 (aged 14–15) pupils. Although the curriculum is targeted at particular year groups, the intervention is a universal ‘whole-school’ intervention and as such has the potential for greater population-level impacts than targeted interventions [9] while minimising the risk of ‘positive deviancy training’, which can be an issue in targeted interventions that bring together ‘at-risk’ individuals [32].

To enable the above intervention components, in the optimisation and pilot stage schools are provided with the following inputs:

1. A manual guiding each of the intervention components
2. The resources to carry out the student survey and compile the student needs data
3. Staff and student training in running a School Health Promotion Council (SHPC), staff training in delivering the social/emotional skills and sex education curriculum, and in running social marketing campaigns, all provided by NCB SEF
4. Curriculum materials in social/emotional skills and sex education provided by NCB SEF
5. Consultation on school sexual health services provided by NCB SEF

### Intervention optimisation

The optimisation of the Positive Choices intervention will be led by the research team and staff from NCB SEF as well as the staff and students of one secondary school plus other youth and policy stakeholders. Optimisation of each of the six intervention components (outlined above) will occur through a systematic process as follows:

1. Review by researchers and NCB SEF staff of existing systematic reviews and the evaluations of and, where appropriate, intervention materials from the Safer Choices, CAS Carrera and Gatehouse interventions
2. Drafting of intervention materials by NCB SEF and the research team
3. Consultation with staff and students from the optimisation school, as well as the other stakeholders
4. Refinement of intervention approaches and materials

### Feasibility assessment and intervention refinement

The intervention components will then be implemented and assessed for feasibility and acceptability in the school involved in optimisation (see ‘Process evaluation’ below). This will occur over one school year in phases as follows:

1. Term 1 (September–December 2017): implementation of student needs survey, staff training and School Health Promotion Council
2. Term 2 (January 2018–March 2018): implementation of student curriculum
3. Term 3 (April 2018–July 2018): implementation of student-led social marketing and consultancy regarding school sexual health services

**Fig. 3:**
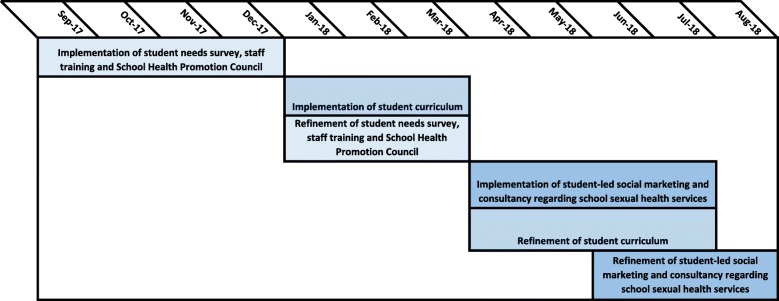
Timeline for feasibility assessment and intervention refinement phase

Intervention components will be assessed by the research team as they are implemented in order to inform further refinements of the intervention components led by NCB SEF (Fig. 3 provides a timeline of the feasibility assessment and intervention refinement phase of the study).

### Pilot cluster randomised controlled trial

The pilot RCT will involve six state secondary schools (four interventions and two controls) in the south-east of England.

### Study settings and population

Positive Choices will be delivered in one English state secondary school in the feasibility phase of the study. In the pilot, six English state secondary schools (including free schools and academies) will be included. Positive Choices is a universal intervention aimed at 11–16-year-olds in participating secondary schools in England. While the intervention will have effects at the whole-school level, the study population in the pilot will be students nearing the end of year 8 (age 12–13 years) at baseline who will receive the year 9 classroom curriculum. These pupils will be at the end of year 9 (age 13–14 years) at follow-up 12 months later (see below). In a phase III trial, the intervention would target students in years 9 and 10. The targeting of year 9 students in the feasibility assessment and pilot RCT phases reflects the truncated timescales of these phases. Years 9 and 10 would be targeted in a phase III RCT because proximal risk factors are manifesting [33], prevention is not too late and sex education is acceptable [34, 35]. Prior stakeholder engagement suggests provision to year 11 students is unfeasible because of GCSE exam preparation.

### Inclusion and exclusion criteria

We will collaborate with one secondary school in the optimisation and feasibility assessment phases. This school will be purposively selected based on location in south-east England and having a higher than median local index of multiple deprivation and value-added GCSE attainment to reflect high need but high capacity to participate in optimisation and refinement.

For the pilot, all state secondary schools (including free schools and academies) in south-east England will be eligible. Private schools, pupil referral units (PRUs) or schools for those with special educational learning needs or disabilities and boys’ (but not girls’) schools will be excluded from the pilot and full trial since our primary outcome focuses on unintended pregnancies among girls.

No students in participating schools will be excluded from our study. Those with mild learning difficulties or poor English will be supported to by fieldworkers to participate in baseline and follow-up surveys (described below).

### Recruitment

The optimisation and feasibility assessment phase of the project will involve one purposively sampled secondary school (see above for criteria) with no random allocation. This school will be recruited via our existing contacts to ensure the school has the capacity to participate.

In the pilot RCT phase, six schools across south-east England will be recruited (purposively varying by local deprivation and school-level GCSE attainment). Schools will be recruited to the pilot RCT by a combination of mail outs, phone calls and prior networks including the UCL Partners School Health and Wellbeing Research Network. Response rates will be recorded, as will any stated reasons for non-participation.

### Randomisation

For the pilot phase, following the baseline survey with students at the end of year 8 (approximately 180 per school), schools will be randomly allocated to intervention/control groups remotely by the Clinical Trials Unit (CTU) at the London School of Hygiene and Tropical Medicine (LSHTM). In the pilot, allocation will be 2:1 favouring the intervention.

### Comparators

In the pilot RCT phase, two schools will be randomised to the control group, will not receive the intervention but will continue with any existing sexual health-related provision, which will be examined in our process evaluation, as will sexual health services in the surrounding area. Retention of control schools will be maximised via £500 payment and feedback of survey data after trial analysis.

### Endpoints of the study

#### Optimisation and feasibility assessment phases

Outcomes for the optimisation and feasibility assessment phases will be meeting criteria for progression to a pilot RCT. Progression criteria comprise the following:

1. Materials for the training, school health promotion council, social marketing meetings, student curriculum and consultancy on school sexual health services are optimised in line with the theory of change and to the satisfaction expressed in writing of the research team, NCB SEF, the participating secondary school and the study steering committee.
2. According to audio-recordings, provider diaries and researcher observations, the training, school health promotion council, social marketing meetings, student curriculum and consultancy on school sexual health service components are implemented with 70% + fidelity in the participating school.
3. Interviews with students and staff conducted as part of the process evaluation (described below) indicate that the intervention is acceptable to at least 70% of students and staff involved in implementation.

Assessment of optimised materials will be based on whether stakeholders agree that intervention materials are consistent with the theory of change, meet the specification laid out in the protocol and are regarded as contextually appropriate for piloting in English secondary schools. Materials will be assessed by the research team and discussed in a minuted investigator meeting. The views of the school on the materials will be assessed as part of the process evaluation via interviews with four school staff and eight year 9 students. The materials, the minutes of the investigator meeting and the findings from the above interviews will then be discussed within a minuted SSC meeting which will make an overall recommendation about whether interventions are acceptable or not and any amendments that are required. Fidelity will be assessed quantitatively against tick-box quality metrics which will form an integral part of each intervention component. For example, each training and curriculum session will be assessed against session-specific quality metrics relating to the topics covered, the exercises used and opportunities for discussion; meetings will be assessed against meeting-specific quality metrics relating to the agenda items covered, opportunities for discussion and the actions agreed; and consultancy on school sexual health services will be assessed against quality metrics concerning the review of existing services and action taken to enhance these. The investigators will agree a set of metrics to assess fidelity and acceptability with the Study Steering Committee (SSC) in the early stages of the project, prior to carrying out fieldwork.

#### Pilot RCT

As already outlined, the pilot RCT will not aim to assess intervention effects, but to assess the feasibility and acceptability of implementing the intervention in state secondary schools in England. Pilot primary outcomes will be meeting criteria for progression to a phase III trial comprising:

1. The intervention is implemented with 70% fidelity in ≥ 3 of four intervention schools.
2. Process evaluation indicates that the intervention is acceptable to 70% of students and staff involved in implementation.
3. Randomisation occurs and ≥ 5 of six schools accept randomisation and continue within the study.
4. Student questionnaire follow-up rates are ≥ 80% in ≥ 5 of SIX schools.
5. Linkage of self-report and routine administrative data on pregnancies is feasible.

#### Measures to be tested as part of the pilot trial

Outcome measures and analyses that would be used in a phase III RCT will be tested in the pilot. Our current intention is that the primary outcome for a phase III trial will examine unintended teenage pregnancies via routine data on births and terminations assessed at 48 months (age 16/17). Around half of under-18 conceptions in England and Wales end in abortion [1]. Yet, while recognising that a proportion of teenage pregnancies will be intended [36], we anticipate that an outcome measure that takes into account both conceptions and terminations will provide a better indication of unintended pregnancies than a measure purely focused on terminations. This is because many unintended pregnancies will not result in termination, and rates of termination will be strongly affected by the availability of local services.

We will also assess secondary outcomes which would be assessed in a phase III RCT at 24 months. The following secondary outcome measures drawn from the Ripple and Share trials [34, 35, 37] will be assessed via self-complete paper and pen questionnaire:

1. Self-reported pregnancy and unintended pregnancy (initiation of pregnancy for boys) and STIs
2. Age of sexual debut, number of sexual partners, use of contraception at first and last sex and non-volitional sex
3. Educational attainment (which is a plausible and, for scale up, critical outcome of our intervention).

The full trial will conduct exploratory analyses to examine how effects on the above outcomes are moderated by SES, gender, ethnicity and baseline risk to assess intervention impact on health inequalities. These analyses will be tested as part of the pilot.

Informed by our theory of change [27–30], we will also conduct exploratory analyses to examine the following mediators [38] using existing measures [34, 35, 39–41]:

1. School-level social norms supportive of positive relationships and sexual health
2. Individual-level sexual health knowledge and skills, contraceptive skills and access, self-efficacy, sexual competence, communication with parents, school engagement and career/educational aspirations

All of the above measures will be assessed for reliability in our pilot. We will assess reliability by reporting an intra-cluster correlation coefficients (ICC) to examine the consistency of measures from baseline to follow-up among the control group (this is a different ICC to that measuring clustering within schools) and Cronbach’s alpha statistics at baseline and follow-up for scaled outcomes. See Economic evaluation section below for economic outcomes.

### Assessment and follow-up

#### Feasibility assessment phase

A baseline needs survey of students approaching the end of year 8 will be undertaken. Other data collected in the feasibility phase are described below under ‘Process evaluation’.

#### Pilot RCT

In the pilot RCT, baseline surveys will be carried out before randomisation as students near the end of year 8 (age 12/13) in June 2018 and will collect data on pre-hypothesised outcome variables, covariates and moderators, drawing on existing survey items as outlined above. Paper questionnaires will be completed confidentially in classrooms supervised by fieldworkers, with teachers remaining at the front of the class to maintain quiet and order, but unable to see student responses. We will survey absent students by leaving questionnaires and stamped addressed envelopes with schools.

We will resurvey students at 12 months (June 2019) as students near the end of year 9 (age 13/14) and will collect self-report data on experiences of the intervention, outcomes and pre-hypothesised potential mediators. Fieldworkers will be blind to allocation. Based on past experience, [37, 42] in the pilot, we expect 95% baseline survey participation and 90% at follow-up. In the pilot, data on terminations and births at 18 months will be obtained in collaboration with the Office for National Statistics (ONS) and the Department of Health (DH) by linking data on female trial participants via the national pupil database and other identifiers to routine ONS data on registration of births and statutory termination notifications, by staff blind to allocation. Linkage of birth and termination data has been previously conducted for observational studies [33] but has not involved linkage to survey data, and hence has not examined participant consent rates, which will be a key focus for our research. Initial discussion with ONS has established that data linkage is feasible despite the limited identifiers attached to termination records and is consistent with DH guidance and data protection law.

### Analytic sample and proposed sample size

Feasibility assessment will be carried out in one London school. Involving only one school at this stage enables intensive engagement while assessing that intervention activities are in principle feasible. The survey will involve approximately 180 year-9 students but note that this is undertaken to assess the feasibility of this as an intervention component only. Evaluation activities (see below for details) in this feasibility phase will involve small samples intended to contribute to assessing feasibility in principle rather than providing statistically representative findings. Qualitative research will involve purposive samples aiming to encompass diversity on key criteria.

Six schools varying by local level of deprivation and school-level GCSE attainment will be recruited to the pilot. No power calculation for this phase has been conducted since the aim of this phase is to assess progression criteria in a pragmatic, relatively small but purposively diverse sample of schools prior to a future phase III RCT which would involve a larger sample in order to examine intervention effects informed by a formal power calculation. Approximately 1080 students will be surveyed in the pilot at the end of year 8 (age 12/13) at baseline and followed-up 12 months later. While no power calculation for this phase has been undertaken, previous similar studies suggest this sample will be sufficient in order to assess student response rates, the reliability of measures and intervention reach [42, 43]. Evaluation activities (see below for details) in this feasibility phase will involve samples intended to contribute to assessing local feasibility rather than providing statistically representative findings. Qualitative research will involve purposive samples aiming to encompass diversity on key criteria.

### Protection against bias

Although the aim of this study is to optimise the intervention, assess feasibility and then pilot outcome measures and analyses rather than estimate intervention effects, we will pilot methods aimed at minimising bias. The investigator team and the intervention delivery team will be separately managed. In the pilot RCT, outcome data will be collected and analysed blind to allocation, and we will examine effects adjusting for potential baseline confounders (age, gender, SES and ethnicity). We will aim to maximise response rates at each pilot RCT site at baseline and follow-up to minimise non-response and attrition bias, for example, following up those individuals not present during survey sessions. Response rates and qualitative data will be analysed to refine data collection methods prior to a phase III trial examining effectiveness. Blinding of participants to allocation is not possible.

### Process evaluation

Integral process evaluation informed by existing frameworks [44–46] has three purposes:

1. To examine intervention feasibility, fidelity, reach and acceptability in the feasibility and pilot RCT phases
2. To assess provision in control schools and potential contamination in the pilot RCT
3. To explore context and potential mechanisms of action in the pilot RCT phase, including potential unintended effects, in order to refine the intervention theory of change and design

#### Feasibility assessment phase

The feasibility assessment phase will assess the ‘progression criteria’ to advance to the pilot RCT phase (outlined above). Data will be collected via audio-recording of NCB SEF training for school staff; surveys of school staff trained by NCB SEF; diaries (including time logbooks) of school staff implementing School Health Promotion Councils, curriculum and social marketing meetings; structured observations of two sessions of School Health Promotion Councils, curriculum lessons and social marketing meetings; and individual or group interviews with four NCB SEF staff and four school staff (purposive by role/seniority) and eight year 9 students (purposive by gender and SES).

#### Pilot RCT phase

In addition to assessing the ‘progression criteria’ relating to intervention feasibility and acceptability (outlined above), we will also examine reach via qualitative research as well as questionnaire survey items at follow-up. The information collected on socio-demographic, educational and neighbourhood characteristics in the student surveys will also allow us to examine reach according to these measures and how this varies by institutional setting. We will also assess the fidelity, reach and perceived impacts of staff training activities. Data will be collected via audio-recording of NCB SEF training for school staff; surveys of school staff trained by NCB SEF; diaries (including time logbooks) of school staff implementing School Health Promotion Councils, curriculum and social marketing meetings; and structured observations of randomly selected session per school of School Health Promotion Councils, curriculum lessons and social marketing meetings. Individual or group interviews with two trainers, four staff per intervention school (purposive by seniority/activity involved), and 8 × year 9 students per intervention school, (purposive by involvement, risk status and gender), will also be conducted.

#### Provision in control schools and potential contamination

We will examine sexual health provision in and around control schools in order to describe our comparator. We will examine the potential for contamination across arms to ensure this is not a threat to internal validity in a phase III trial. Data will be collected via student surveys, interviews with two staff per control school (purposive by seniority) and 4 × year-9 students (purposive by gender and SES) per control school.

#### Context and mechanisms of action

In addition to piloting intermediate outcome variables required for mediator analyses in a subsequent phase III RCT, we will use rich, contextual qualitative data and analyse these data in order to explore potential mechanisms of action and thus refine our theory of change. These qualitative analyses will also examine how mechanisms may vary with context, students’ socio-demographic characteristics and/or other factors, in order to refine and optimise the intervention’s theory of change. We will also analyse qualitative data to explore any mechanisms that might give rise to unintended, potentially harmful consequences.

### Economic evaluation

The pilot RCT will examine whether it is feasible to assess cost-effectiveness using a cost-consequence analysis within a phase III trial. Within the pilot, study methods to measure the incremental cost of the intervention in a phase III trial study will be developed and piloted. With the use of a broad public and third sector perspective, resources to be measured will include resources used by NCB SEF, schools and the NHS. Within this, key interventional resources will include NCB SEF and school staff time, training events/workshops and consumables. Measures will include standardised sessional checklists to monitor and document attendance, preparation and delivery time for key training events, School Health Promotion Councils, student-led social marketing meetings and the review of school sexual health services; the completion of surveys and diaries by school staff charged with intervention delivery, assessing time spent on tasks relating to intervention, staff travel and other expenses relating to the intervention charged to a specific project grant code.

The Child Health Utility (CHU) 9D measure [47] will be used to assess student’s health-related quality of life as part of the economic evaluation. The CHU-9 is a validated age-appropriate measure that was explicitly developed using children’s input and has been suggested to be more appropriate and function better than other health utility measures for children and adolescents. Student utility values will be collected (at baseline and at follow-up surveys at 24 and 36 months in a full RCT) using the CHU-9D and by converting the SF-12 questionnaires, respectively. It is anticipated that these measures would be used in a phase III trial to measure short-term impact on health-related quality of life.

### Analyses

#### Feasibility assessment

Our analysis in this phase will determine whether the study should proceed to the pilot RCT phase. Descriptive statistics on fidelity will draw on audio-recordings of training, diaries of providers and structured observations of intervention activities. Analysis of acceptability will draw on interviews with staff and students. Findings will be fed back to NCB SEF staff who will be responsible for refining the intervention ready for implementation in the pilot RCT.

#### Pilot RCT

Our main analyses will determine whether criteria for progression to a phase III trial are met. Descriptive statistics on fidelity will draw on audio-recordings of training, diaries of providers and structured observations of intervention activities. Statistics on acceptability will draw on surveys of students and trained staff and interviews with staff and students. School randomisation and retention, and student follow-up will be described using a CONSORT diagram [48]. We will assess the precision of data linkage in association with ONS researchers.

Additional analyses will address our other research questions. Descriptive summaries of baseline and follow-up data by arm will be tabulated. We will assess the reliability of secondary outcome measures by reporting intra-cluster correlation coefficients (ICC) to examine the consistency of measures from baseline to follow-up (this is different from the use of ICC to measure clustering of variables within schools) and Cronbach’s alpha statistics at baseline and follow-up for scaled outcomes. We will pilot intention-to-treat analyses of outcomes [44] and moderator analyses (how effects vary by SES, gender, ethnicity and baseline risk).

Qualitative data will be subject to thematic content analysis (in vivo/axial codes; constant comparison [49] informed by realist approaches to evaluation [50] and May’s implementation theory [46] to examine potential mechanisms of action and of harm, determine how contextual factors influence implementation and mechanisms, describe relevant activities in and around intervention and control schools and refine our programme theory and theory of change.

Our economic feasibility study will pilot collection of quality of life and assess the feasibility of methods to be used within a full trial, which in line with NICE guidance, would involve a wider cost consequence analysis, comparing intervention costs with the full range of study outcomes.

### Ethical issues

Ethical approval for the study was obtained from the London School of Hygiene and Tropical Medicine Ethics Committee on 21 March 2017 (Ref. 11927).

#### Informed consent

Head teachers as gatekeepers will be asked for informed consent for intervention and random allocation, as is standard practice in cluster randomised trials in schools [42]. As is normal within public health and educational research in secondary schools in the UK (e.g. [34, 35, 37]) informed written opt-in consent will be sought from all research participants, including students, judged competent to provide this (a model information sheet and consent form is provided at Additional file 1). In all cases of data collection including surveys, interviews and focus groups, observations and audio-recordings, except where practically impossible, participants will be given an information sheet several days before data collection. Just before data collection participants will also receive an oral description of the study and have the opportunity to ask questions. Participants will then be advised that participation is voluntary, and they may withdraw at any point. All participants will be advised that they are free to withhold consent, and this matter will not be fed back to teachers or, in the case of staff participants, their managers. Students opting not to participate in surveys will be offered alternative activities in the classroom. Those opting out of other data collection will be free to continue with their normal activities. All participants, including students, will be informed in consent materials of the confidentiality with which the information they provide will be treated as well as the circumstances in which we would need to breach confidentiality (see ‘Safeguarding’ below).

The research will also involve the piloting of the linkage of student survey data to administrative data on births and terminations by the Office for National Statistics. Survey participants will be informed of this process as part of consent procedures and their consent to it sought.

In addition, students’ parents will be contacted by letter 1 week prior to any specific research fieldwork informing them about this and providing them with the option of withdrawing (opting out) their child by contacting the school or the research team. As is normal within public health and educational research involving secondary school students in the UK, we will not seek opt-in consent from student participants’ parents.

#### Safeguarding

We will develop and maintain standard operating procedures for dealing with safeguarding concerns and reporting serious adverse events. In collaboration with the National Children’s Bureau, we will develop a priori categories of abuse reported through the research that necessitate our breaching confidentiality to ensure individuals are offered care and protection. These criteria will be established so that we balance our ethical duty of promoting participant autonomy by respecting confidentiality and promoting participant wellbeing when we determine that we need to breach confidentiality to address abuse that appears to be serious and ongoing. Where such abuse is reported through a questionnaire, we will contact the safeguarding lead in the school. Where the report occurs directly to research staff, we will first discuss the need for a response with the research participant prior to contacting the school safeguarding lead.

Qualitative research (interviews, focus groups, observations) will not ask staff or students about their experience of sex. However, if participants nonetheless describe any sexual abuse, or otherwise become upset in any way, our researchers will be trained in how to respond. In the case of focus groups, researchers will be trained to ensure that discussions do not move in the direction of personal disclosures of sexual behaviour since this is not the purpose of the groups and it would be very difficult to ensure that all focus group participants did not talk about such disclosures outside the group. Staff will be trained to identify the potential for such disclosures, work to avoid them but then to approach participants immediately after the focus group to offer support and to assess whether any other response is needed, using the same procedures as described above.

Any member of the research/fieldwork team visiting a school will be required to have a full Disclosure and Barring Services (DBS) check. All work will be carried out in accordance with guidelines laid down by the Economic and Social Research Council (ESRC), the Data Protection Act 1998 and the latest Directive on GCP (2005/28/EC).

The trial steering group (which because this is a pilot not a phase III RCT will undertake data monitoring and ethics duties) and the London School of Hygiene and Tropical Medicine ethics committee will be provided with anonymised reports of all disclosures of serious abuse and any other serious adverse events. These will categorised by type, circumstances and the extent of any possible connection with intervention or research activities.

In each school and within NCB a senior member of staff will be identified who is not directly involved with the intervention and whom staff or students may go to if they have complaints about any elements of the research study. This will be communicated to students outside of the research process to increase trust that this is truly independent.

Quantitative and qualitative data will be managed by project staff using secure data management systems and stored anonymously using participant identification numbers. Quantitative data will be managed by London School of Hygiene and Tropical Medicine accredited clinical trials unit (CTU). Where collected, participant identification numbers and corresponding participant names will be held in separate files; these files will be password-protected folders. The names used in qualitative data will be replaced with pseudonyms in interview/focus group transcripts. In reporting the results of the process evaluation, care will be taken to use quotations which do not reveal the identity of respondents.

In line with MRC guidance on personal information in medical research, we will retain all research data for 20 years after the end of the study. This is to allow secondary analyses and further research to take place and to allow any queries or concerns about the conduct of the study to be addressed. In order to maintain the accessibility of the data the files will be refreshed annually and upgraded if required.

### Public and patient involvement (PPI)

In preparing this protocol, we have collaborated with staff and students from five schools involved in UCL Partners Schools Health Research Network (co-directed by CB) via consultations in September–October 2014. These informed our decisions to focus our year 9 curriculum on social/emotional skills and our year 10 curriculum on sexual health and contraception/protection, include a focus on school sexual health services, ensure student-led social marketing embraces social media, use interviews where appropriate in our process evaluation and interview students as well as staff in control schools to assess usual provision.

We also consulted with five members of the ALPHA (Advice Leading to Public Health Advancement) youth group based at the DECIPHer Centre, Cardiff University, on 29 October 2014. Participants were enthusiastic about the intervention, supported this starting in year 9, very supportive of school-based sexual health services and felt that targeting would be problematic. Although some components are already being delivered in some schools, none use a coherent programme informed by social marketing principles.

Ongoing consultation with NCB SEF and one secondary school in London is inbuilt into the optimisation phase of the study. Policy stakeholder’s events and consultation with the ALPHA youth advisory group will continue to take place throughout the duration of the project.

### Expected output of research/impact

As well as reporting in the NIHR Public Health Research journal, we would submit two open-access papers to high-impact journals reporting our key findings regarding (1) process evaluation of integrated social marketing strategy and (2) student/staff experiences of the intervention. We will present our findings at two international conferences (Society of Prevention Research and International Association for Adolescent Health) in 2019, as well as national conferences. We will disseminate the results to participating schools, to the ALPHA youth group based at DECIPHer and to schools in the Institute of Education/UCLPartners School Health and Wellbeing Research Network and Healthy Schools London network, both of which we are already heavily involved in. We will draft an article for the Times Education Supplement about the research. The research team will also use blog-posts and Twitter to increase public awareness of the study. Knowledge exchange is built into the proposed work from the outset via the stakeholder group. We will present emerging findings at two meetings with policy stakeholders, including policy officials and public health commissioners in the UK nations. Two policy and practice dissemination events will be held: one seminar in partnership with Public Health England and one at the Association for Young People’s Health.

The most important scientific outputs generated by this project will be increased knowledge about the feasibility and acceptability of delivering and trialling an intervention which uses social marketing strategies and is informed by existing effective interventions to prevent unintended teenage pregnancies. This will inform the development of a subsequent proposal for a phase III effectiveness trial.

### Research governance

#### Trial registration and conduct

The trial is registered with www.controlled-trials.com (ISCTN 12524938). As the trial does not take place within clinical settings nor using clinical samples nor using a medicinal product, there is no requirement to comply with the ‘The Medicines for Human Use (Clinical Trials) Regulations 2004’. We will follow the UK Medical Research Council (MRC) guidelines on good clinical practice for clinical trials.

The London School of Hygiene & Tropical Medicine will act as the main sponsor for this study. Delegated responsibilities will be assigned locally.

The principal investigator (CB) will have overall responsibility for the conduct of the study. The day-to-day management of the trial will be coordinated by the trial manager (RP) based at LSHTM. The following governance structures will be instituted:

1. *Trial executive group (TEG):* the PI (CB) will chair weekly TEG meetings with the trial manager (RP), statistician (EA) and, where appropriate, NCB SEF, CTU and fieldwork staff.
2. *Trial investigators’ group (TIG*): CB will also chair a TIG which will include all co-investigators and members of the TEG; the TIG will meet monthly during the early stages of the research (months 1–6) and then every 3 months thereafter.
3. *Study steering committee (SSC)*: an independent SSC will be established and meet three times throughout the life of the project to advise on the conduct and progress of the trial, and relevant practice and policy issues. Because this is a pilot not a phase III RCT, the SSC will undertake data monitoring and ethics duties and be informed of any serious adverse events as described under ‘ethics’ above.

The project will employ standardised research protocols and pre-specified progression criteria, which will be agreed and monitored by the TIG and SSC.

## Discussion

This will be the first UK-based pilot trial of a school-wide social marketing intervention to reduce unintended teenage pregnancy and improve sexual health. The study takes an innovative approach, encompassing the systematic optimisation of the Positive Choices intervention, feasibility testing and refinement in one school followed by a pilot trial in six schools and accompanied with integral process evaluation that draws on principles of realist evaluation to understand processes and mechanisms of action. If the pilot trial proves feasibility and acceptability of the optimised Positive Choices intervention in English secondary schools, plans will be initiated for a phase III trial of intervention and cost-effectiveness. As a universal whole-school social marketing intervention, if found to be effective, Positive Choices has the potential to make significant population-level health improvements.

### Trial status

At the time of submission (November 2017), the optimisation school has been recruited, the needs survey has been carried out in this school, the first set of intervention materials have been optimised and implementation of the intervention for the feasibility assessment phase has begun. The research team is in the process of recruiting schools for the pilot trial.

## Additional file

Additional file 1: Consent form for ‘Positive Choices’ student questionnaire. (PDF 140 kb)
