# Supplementary material for: Study protocol for the optimisation, feasibility testing and pilot cluster randomised trial of Positive Choices: a school-based social marketing intervention to promote sexual health, prevent unintended teenage pregnancies and address health inequalities in England
Source: Pilot Feasibility Stud. 2018 May 23;4:102. doi: 10.1186/s40814-018-0279-3 (PMC5964886; doi:10.1186/s40814-018-0279-3)
Supplement: Supplementary file 1 — Consent form for ‘Positive Choices’ student questionnaire. (PDF 140 kb) [file 40814_2018_279_MOESM1_ESM.pdf]

Consent form for ‘Positive Choices’ student questionnaire

We are researchers from the London School of Hygiene and Tropical Medicine. We are working with the National Children’s Bureau Sex Education Forum (NCB SEF) and your school to develop and test out a new sex and relationships education programme for secondary schools in England called ‘Positive Choices’. As part of our research, we are asking all Year-8 students at Lambeth Academy to fill in a questionnaire at school. The questionnaire takes about 45 minutes to complete. It will be carried out in a classroom or the school hall and will be supervised by a member of school staff. A trained researcher will be present to explain and answer any questions about the questionnaire, and make sure you have the peace and privacy you need to fill it in. The answers you give will be used to inform how we develop and tailor our sex and relationships programme for your school.

The questionnaire includes questions about your views on sex education; your knowledge and attitudes about sexual heath; your experiences of sexual harassment at school; your experiences of seeing sexual imagery; and communicating with parents about personal issues. If anything in the questionnaire causes you to feel confused or concerned you should speak to a trusted adult such as a parent/carer or member of staff at your school. You may also ask to speak privately to the researcher. The information sheet you received recently also included a list of people and organisations you can contact for support both inside and outside school if you have any questions about sex and relationships or want speak with someone about any issue you (or a friend) are going through. Another copy has been given to you today to take away with you.

The questionnaire is <u>completely anonymous and totally confidential.</u> This means that <u>your name will not be connected to your answers</u>. Anything you report in the questionnaire will be kept private. We will not share it with other people such as teachers and parents. We will securely store answers to the questionnaire in our offices on a computer database that only the research team can access. The file will <u>not include, and cannot be linked to, your name or any way of identifying you</u>. When we write research reports or articles based on the answers from the questionnaires, you and your school will not be <u>named or in any way identified</u>. Your school will receive a report for the staff and students describing our findings, but there will be no way to link what individual students report with the overall findings in the school.

You can decide whether or not to take part in our research. If you do choose to take part, you may stop taking part at any time with no negative consequences – it is completely up to you.

LONDON SCHOOL of HYGIENE &TROPICAL MEDICINE

If you are happy to fill in the questionnaire, please fill in the box below. This consent form will be kept separately from the questionnaire and will not be linked to your answers to the questionnaire.

Full Name ……………………………………………………..…………..

I have read the information above.

I have been given the opportunity to ask questions and have them answered.

I understand that I can choose to take part or not.

I understand that I can stop taking part at any time.

<u>I agree to take part in this study.</u>

Signed ……………………………. Date ……………………………………..
